# The Evaluation of the Comparision Between pre- and post Pandemic Era Regarding Emergency Psychiatric Consultations

**DOI:** 10.1192/j.eurpsy.2022.1251

**Published:** 2022-09-01

**Authors:** V. Çakır Kardeş, G. Telli

**Affiliations:** Zonguldak Bülent Ecevit University, Faculty of Medicine, Department Of Psychiatry, Zonguldak, Turkey

**Keywords:** COVID-19, emergency psychiatry consultations, pandemic

## Abstract

**Introduction:**

COVID-19 had direct and indirect impacts on both mental health and healthcare systems. Evaluating urgent psychiatric consultations may be useful to determine the effects of COVID-19 pandemic since it reflects the condition of psychiatric patients and healthcare systems

**Objectives:**

This study aims to determine the quantitative or qualitative changes in emergency psychiatry consultations after COVID-19 pandemic.

**Methods:**

The socio-demographic characteristics and clinical features of two hundred thirty three patients were retrospectively collected and analyzed in order to compare the emergency psychiatry consultations before (between the dates 11^th^ of March 2019-10^th^ of March 2020) and after (between 11^th^ of March 2020-10^th^ of March 2021) the COVID-19 pandemic.

**Results:**

The ratio of patients consulted to psychiatry to total emergency department increase after pandemic (%0.03 vs %0.07). Among these patients, the diagnosis of ‘alcohol and substance use disorder’ (%6.1 vs. %15.4) (p=0.03) increased while the diagnoses of ‘obsessive compulsive disorder (5.3% vs. 0%)(p=0.01) and bipolar disorder (%21.1 vs. %20.5) (p=0.02) decreased. Hostility among patient during consultation increased (%19.1 vs. %30.8)(p=0.04). Suicidal thoughts decreased (%25.2 vs. %14.5) (p=0.04). Furthermore, voluntary inpatient treatment (%20.9-%34.2) (p =0.02) increased, transfer to another clinic (%25.2 vs. %12) (p=0.01) and outpatient treatment (%46.1 vs. %42.7) (p=0.01) decreased. An increase in oral treatments (%10.4 vs. %26.5) (p=0.02) and decrease in parenteral treatments (%71.3 vs. 54.7) ( p=0.01) were also reported.

**Conclusions:**

Our findings confirmed that after COVID-19 spread the clinical features diagnosis, and treatment modality have changed among urgent psychiatric consultations.

**Disclosure:**

No significant relationships.

